# Dysregulated lncRNAs are involved in the progress of myocardial infarction by constructing regulatory networks

**DOI:** 10.1515/med-2023-0657

**Published:** 2023-03-08

**Authors:** Jingqi Yang, Ming Yang, Guotai Sheng

**Affiliations:** Department of Cardiovascular Medicine, Jiangxi Provincial People’s Hospital, The First Affiliated Hospital of Nanchang Medical College, Nanchang, 330000, China

**Keywords:** long noncoding RNA, myocardial infarction, immune system, inflammation, regulatory networks

## Abstract

Long noncoding RNAs (lncRNAs) mediate important epigenetic regulation in a wide range of biological processes. However, the effect of all dysregulated lncRNAs in myocardial infarction (MI) is not clear. Whole transcriptome sequencing analysis was used to characterize the dynamic changes in lncRNA and mRNA expression. A gene network was constructed, and genes were classified into different modules using WGCNA. In addition, for all dysregulated lncRNAs, gene ontology analysis and *cis*-regulatory analysis were applied. The results demonstrated that a large number of the differentially co-expressed genes were primarily linked to the immune system process, inflammatory response, and innate immune response. The functional pathway analysis of the MEblue module included immune system process and apoptosis, and MEbrown included the T-cell receptor signal pathway by WGCNA. In addition, through *cis*-acting analysis of lncRNA regulation, the *cis*-regulated mRNAs were mainly enriched in immune system processes, innate immune responses, and VEGF signal pathways. We found that lncRNA regulation of mRNAs plays an important role in immune and inflammatory pathways. Our study provides a foundation to further understand the role and potential mechanism of dysregulated lncRNAs in the regulation of MI, in which many of them could be potential targets for MI.

## 1 Introduction

Long noncoding RNAs (lncRNAs) are noncoding RNAs whose transcript length exceeds 200 nt [1]. Many lncRNAs have 5′capping, alternative splicing, and polyadenylation, similar to mRNAs [2]. Despite their similarity, lncRNAs are regulated in different ways and have a wide range of functions in many physiological and pathological contexts [3]. Most lncRNAs are poorly conserved, and their expression levels are significantly lower than those of mRNAs [4]. The expression patterns are tissue and stage specific, especially for cellular differentiation and development [5,6].

Myocardial infarction (MI) is a disease caused by coronary artery plaque rupture, thrombus blocking blood vessels, and finally causing acute myocardial ischemic injury, which has become one of the main reasons that seriously threatens human health [7]. At present, the treatment for MI mainly includes percutaneous coronary intervention combined with antiplatelet and/or anticoagulation therapy to complete revascularization and lipid regulation to stabilize plaque [8], but the morbidity and mortality of MI are still high. Therefore, further research on the pathogenesis of MI is of great significance to find new molecular targets for diagnosis and treatment.

After MI, myocardial cell necrosis triggers an inflammatory response, which not only participates in the repair of the infarcted myocardium but also participates in the enlargement of the infarct and the aggravation of fibrosis, thereby causing adverse ventricular remodeling [9]. Endothelial cells are a major source of proinflammatory chemokines after MI [10]. Natriuretic release from infarcted cardiomyocytes activates the endothelial inflammatory phenotype and mediates the adhesion of circulating leukocytes to promote leukocyte recruitment. Under the induction of chemokines, complement and interleukins, a large number of leukocytes, especially neutrophils, infiltrate the infarcted myocardium [11]. It has been reported that neutrophils may exert cytotoxic effects on living cardiomyocytes damaged by ischemia in the infarct marginal zone [12]. In addition, after MI, necrotic cells trigger innate immune pathways and activate a range of inflammatory mediators, including inflammatory cytokines, chemokines, and cell adhesion molecules. Therefore, immune and inflammatory responses have an important impact on MI.

In recent years, with the deepening of the study of lncRNAs, circulating plasma or serum lncRNAs, as new biomarkers, have played an important role in disease diagnosis, prognosis, or treatment. In addition, several lncRNAs have been shown to be directly related to MI. For instance, Li et al. [13] used a gene chip to explore the difference in the expression of lncRNAs between MI cells and normal cells and found that 323 lncRNAs were differentially expressed, of which 168 were upregulated and 155 were downregulated. By analyzing the function of lncRNAs with dysfunctional expression, it is predicted that these lncRNAs may promote the occurrence and development of MI. It was found that the expression of lncRNA AZIN2-sv was upregulated in cardiomyocytes and can induce cardiomyocyte apoptosis and reduce cardiomyocyte proliferation, thus inhibiting angiogenesis [14]. Further experiments showed that silencing AZIN2-sv expression can promote myocardial capillary formation after MI, help to increase the left ventricular ejection fraction and left ventricular shortening fraction, and effectively improve the prognosis of MI in rats. Many studies have been conducted on the function of lncRNAs in MI, but most of them focus on a particular lncRNA, and the functions of some lncRNAs are still unclear [15,16,17]. To date, our study is the first to investigate the function of dysregulated lncRNAs in MI by transcriptome sequencing. This article mainly analyzed the gene expression network in the process of lncRNAs regulating the occurrence and development of MI to analyze the overall differentially expressed lncRNAs in MI and their functions.

## 2 Materials and methods

### 2.1 Retrieval and processing of public data

The RNA-seq data of GSE114695 from the GEO database in the present study were obtained. GSE114695 included the mouse left ventricle at 1 day (1 D) and 1 week (1 W) after MI or sham operation. MI was induced by permanent ligation of the left anterior descending coronary artery in 8-week-old male mice. A total of four sets of data were used in our study, including 1 D and 1 W after MI and then 1 D and 1 W after sham operation, and each set had three samples. The proportion of mapping of each sample was more than 80%, and the correlation in the group was better with no group outliers.

SRA Run files were converted to fastq format using NCBI SRA Tool fastq-dump. The raw reads were trimmed of low-quality bases, and clean reads were evaluated using FASTX-Toolkit (v.0.0.13; http://hannonlab.cshl.edu/fastx_toolkit/) and FastQC (http://www.bioinformatics.babraham.ac.uk/projects/fastqc).

### 2.2 Read alignment and differentially expressed gene (DEG) analysis

The obtained high-quality sequence data were aligned with the mouse reference genome (GRCm38) using HISTA2, and the counts of each gene were counted by the feature Counts (1.5.0-p3) tool in Subread software [18]. The expression levels of genes were evaluated using per kilobase of exon per million fragments mapped (RPKM). The DEGs were counted by DESeq2 (3.18.1) [19] with log_2_ fold change >1 (upregulated) or ≤−1 (downregulated) and false discovery rate ≤0.01.

### 2.3 lncRNA prediction and direction identification

**Figure 1:**
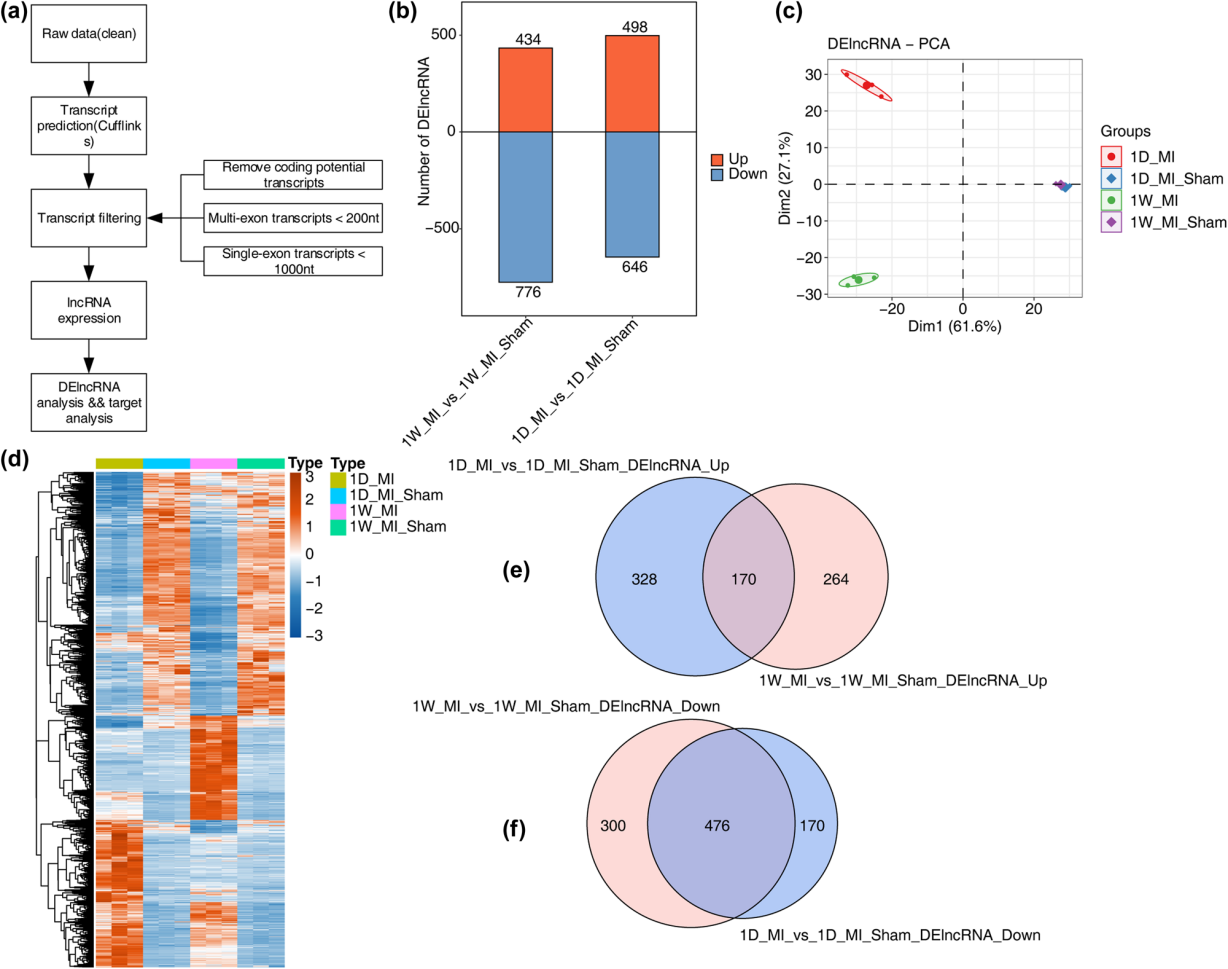
Genome-wide profiling of MI-associated lncRNAs. (a) Illustration of the experimental design and bioinformatics analysis pipeline for the identification and functional annotation of lncRNAs expressed in MI and sham samples. (b) The number of differentially expressed lncRNAs (DE lncRNAs) among the 1-day (1 D) and 1-week (1 W) groups. The number of upregulated and downregulated DE lncRNAs is shown in the bar plot. (c) PCA of MI and sham samples based on the normalized expression level. The samples were grouped by disease state, and the ellipse for each group is the confidence ellipse. (d) Expression heatmap of DE lncRNAs among MI and sham samples. (e and f) Venn diagram of DE lncRNAs in MI and sham samples at 1 day (1 D) and 1 week (1 W).

To systematically analyze the lncRNA expression pattern and methods, we used a previously reported lncRNA identification method based on cufflink software [20,21]. All steps of the analytical methods are shown in Figure 1a. We calculated the coding potential score to filter the coding potential transcripts. When filtering the single-exon lncRNAs, we set the threshold to 1,000 nt for filtering the single-exon lncRNAs and 200 nt for filtering the multiexon lncRNAs.

### 2.4 WGCNA and coexpression analysis

To comprehensively understand the gene expression patterns, weighted gene coexpression network analysis (WGCNA) [22] was used to cluster genes with similar expression patterns and default parameters. All expressed genes in the RNA-Seq data were used as input data. To analyze the regulatory mode between lncRNAs and mRNAs, we calculated the Pearson correlation coefficients and divided their relationship into three categories according to the Pearson correlation coefficient value: positive correlation, negative correlation, and noncorrelation.

### 2.5 Functional enrichment analysis

To analyze the functional classes of DEGs, the KOBAS 2.0 server was used to perform Gene Ontology (GO) terms and KEGG pathways [23]. In addition, the sets of selected genes were identified using Reactome (http://reactome.org) pathway profiling [24].

### 2.6 *Cis* acting

Correlation coefficients and *P* values between mRNA-lncRNAs were obtained based on the expression of each mRNA and the differentially expressed lncRNAs. Then, we filtered through a threshold with an absolute correlation coefficient of not less than 0.6 and a *P*-value of less than 0.05 to form an expression network. For each differentially expressed lncRNA, we obtained the expressed genes within 10,000 bases from its upstream and downstream regions that overlapped with coexpressed genes to obtain lncRNA targets.

### 2.7 Other statistical analyses

Principal component analysis (PCA) was carried out by factoextra (http://www.sthda.com/english/rpkgs/factoextra) in the R package to show the clustering between each sample using RPKM of all DE lncRNAs. After the readings of each gene in the sample were normalized by TPM (Tags Per Million), the internal script was used for the visualization of next-generation sequence data and genome annotations. The pheatmap package (https://cran.r-project.org/web/packages/pheatmap/index.html) in R was used to perform clustering based on Euclidean distance. Student’s *t*-test was used for comparisons between two groups.

### 2.8 Identification of differentially expressed coexpression-related genes

The GSE59867 dataset, based on the GPL6244 platform, was published by Maciejak et al. [25] and was used as the independent external validation set, including 111 patients with AMI at admission and 46 patients with stable coronary artery disease. The “limma” package was used to normalize the gene expression profiles [26].

Whole blood samples were collected from five AMI patients and five normal patients for real-time quantitative polymerase chain reaction (qPCR) to confirm the results. The study was approved by the Ethics Committee of Jiangxi Provincial People’s Hospital, and all patients signed informed consent forms. All patient samples were processed to isolate peripheral blood mononuclear cells immediately after collection and stored at −80°C before RNA extraction. After the samples were pretreated, RNA was extracted using TRIzol reagent (Invitrogen), and qPCR was performed. Total RNA was reverse transcribed into complementary DNA by a qPCR real-time kit (Invitrogen) following the manufacturer’s instructions. Relative gene expression was analyzed by the 2^−ΔΔCT^ method with normalization to ACTB (internal reference gene). All primers used in this study are shown in Table 1. Data are presented as the mean ± standard deviation. GraphPad Prism 8 software (GraphPad Software, CA) and R software were used for statistical analyses. Analysis of variance or a *t* test was used for statistical comparisons. *P* < 0.05 was considered significant.

**Table 1:** Primer sequences

	Forward primer (5′–3′)	Reverse primer (5′–3′)
Isg20	TCTACGACACGTCCACTGACA	CTGTTCTGGATGCTCTTGTGC
Myd88	GGCTGCTCTCAACATGCGA	CTGTGTCCGCACGTTCAAGA
Ecm1	AGCACCCCAATGAACAGAAGG	CTGCATTCCAGGACTCAGGTT
Irf7	CCCACGCTATACCATCTACCT	GATGTCGTCATAGAGGCTGTTG
Ecsit	AACCTCTACTACCCGATGCAG	CAGCCACTCCTCACATACTCC
C3	GGGGAGTCCCATGTACTCTATC	GGAAGTCGTGGACAGTAACAG

## 3 Results

### 3.1 Workflow

We downloaded the RNA-seq sample (GSE114695) of the MI mouse model. The cDNA library was constructed by using the mouse left ventricle at 1 day (1 D) and 1 week (1 W) after MI. The transcriptome data of 12 mRNAs in two different stages of MI (1 D and 1 W) were generated and differentially expressed lncRNA analysis and lncRNA‒mRNA network analysis were carried out. The analysis process is shown in Figure 1a.

### 3.2 lncRNAs were differentially expressed in MI

To identify possible lncRNAs participating in MI, the differentially expressed lncRNAs at 1 day and 1 week after MI were analyzed by RNA-seq. The results showed that compared with the sham-operated group, 1,210 lncRNAs were significantly differentially expressed in the first week, of which 434 were upregulated and 776 were downregulated, and on the first day, 1,144 lncRNAs were significantly differentially expressed, including 498 upregulated and 646 downregulated lncRNAs, which were visualized with a bar plot (Figure 1b). For 1 day and 1 week after MI, through PCA, it was found that the quality of this dataset was good, and the differentially expressed lncRNAs could clearly distinguish the samples of the MI group from the sham operation group (Figure 1c). The differentially expressed lncRNAs between the MI group and the normal group were visualized by heatmap at 1 day and 1 week after MI (Figure 1d). Through the Venn diagram, it was found that 170 lncRNAs were upregulated at both 1 day and 1 week, and 476 lncRNAs were downregulated at both 1 day and 1 week (Figure 1e and f).

### 3.3 Characteristics of lncRNAs detected in MI and Sham samples

**Figure 2:**
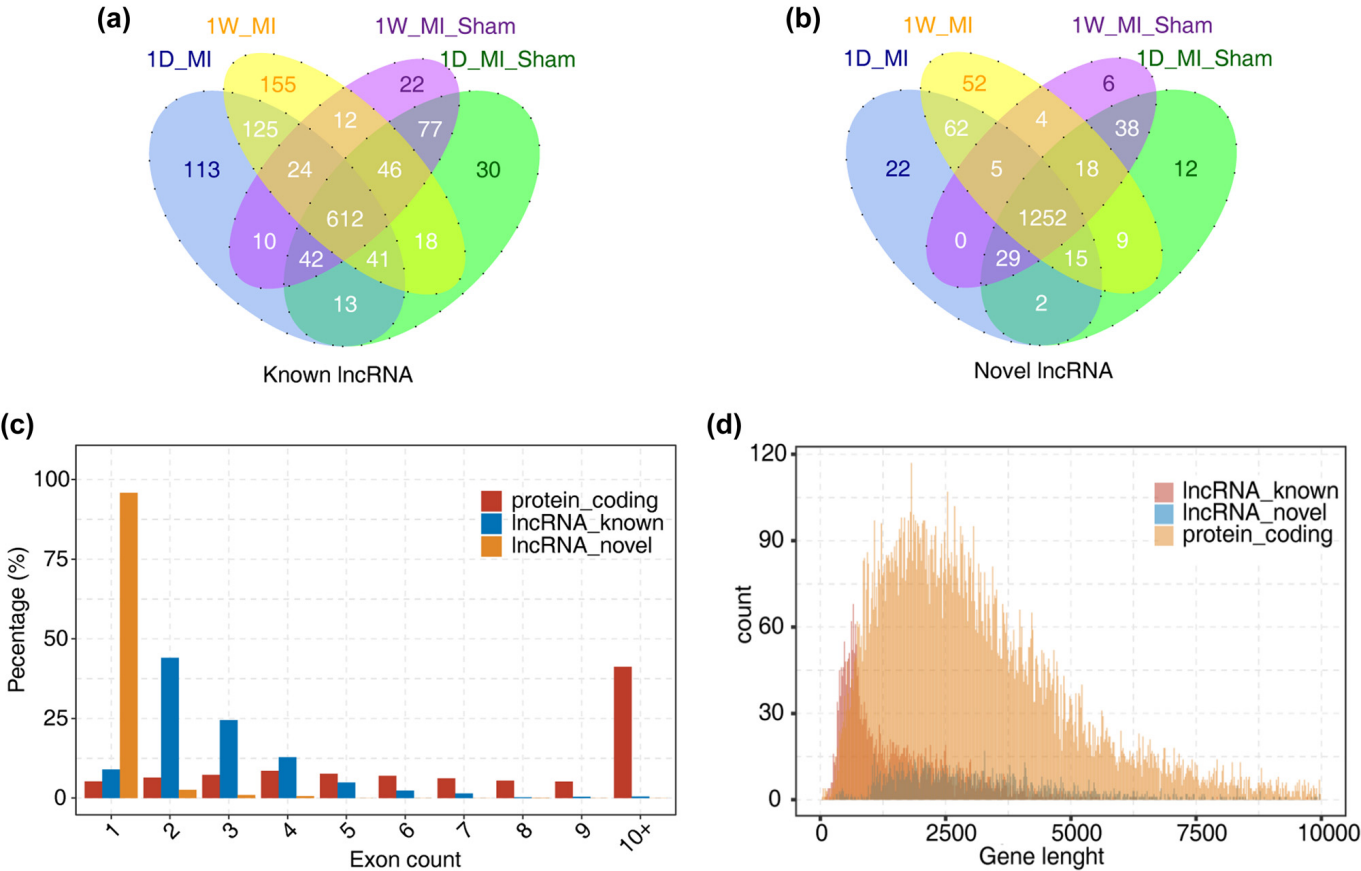
Characteristics of lncRNAs detected in MI and sham samples. Venn diagram of detected known lncRNAs (a) and novel lncRNAs (b) in MI and sham samples. lncRNAs that were detected (RPKM ≥ 0.2) in at least two samples were considered to be detected in the group. (c) Distribution of exon counts of known lncRNAs, novel lncRNAs, and protein-coding RNAs. (d) Density of the length distribution of known lncRNAs, novel lncRNAs, and protein-coding RNAs. The length density distribution was generated by the density function in R.

At present, the functions of many known lncRNAs have been gradually clarified, and some novel discovered lncRNAs are still unclear. To explore the overall function of lncRNAs, we detected known and novel lncRNAs in MI and sham samples after 1 day and 1 week (Figure 2a and b). Most of the novel lncRNAs had only 1 exon, and the gene length of lncRNAs was also shorter than that of mRNAs. The characteristics of lncRNAs are shown in Figure 2c and d.

### 3.4 Construction of the lncRNA‒mRNA coexpression network

**Figure 3:**
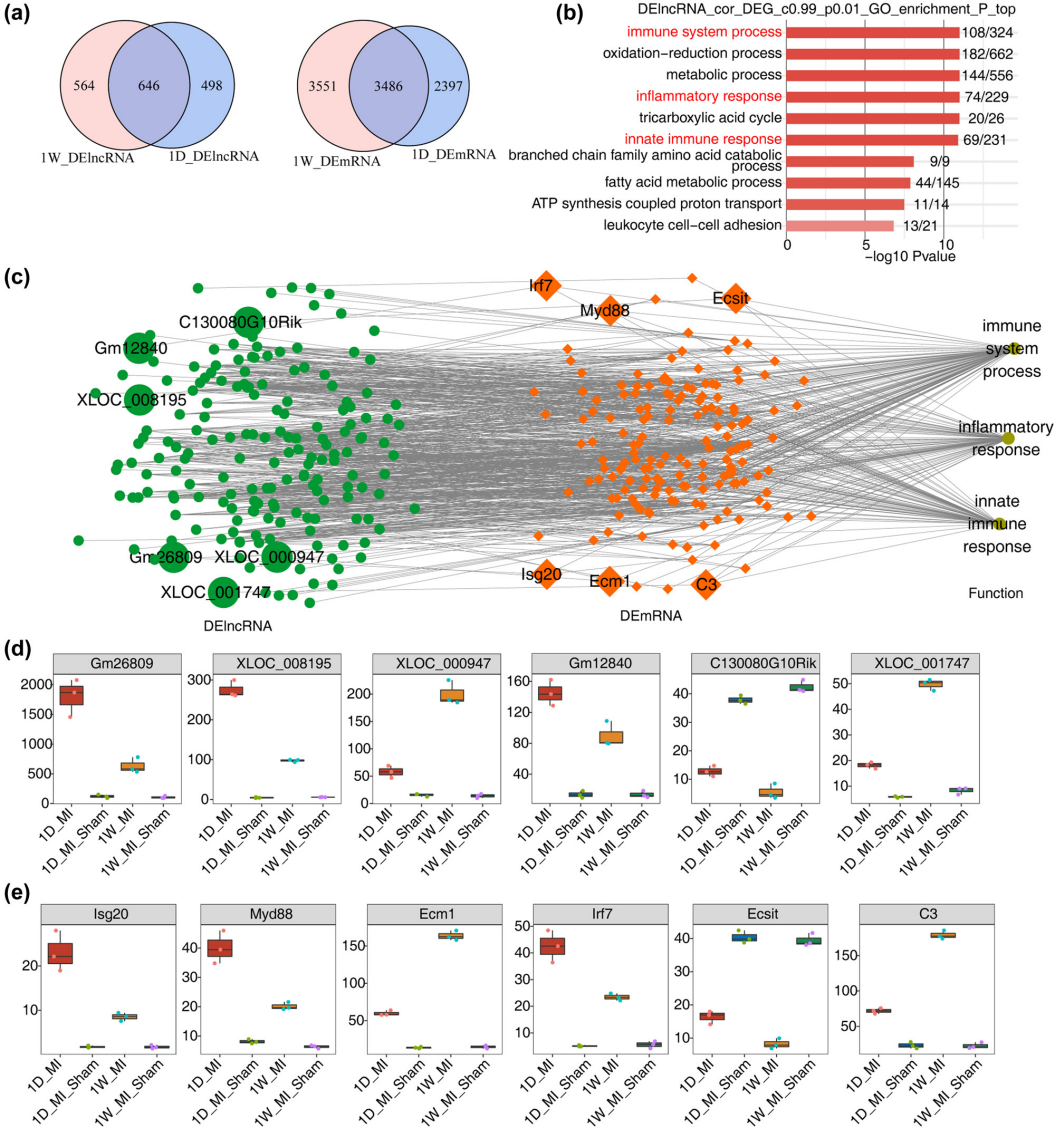
Coexpression network illustration between DE lncRNAs and DE mRNAs. (a) Venn diagram of DE lncRNAs and DE mRNAs in MI and sham samples; (b) The top 10 enriched GO biological process pathways by DE lncRNAs coexpressed with DE mRNAs of MI compared with sham samples. (c) The coexpression network between DE lncRNAs and DE mRNAs involved in the top 3 immune-related terms shown in (b). lncRNAs are on the left, coexpressed mRNAs are in the center, and the mRNA-enriched GO terms are on the right. (d and e) Boxplots showing the expression of six DE lncRNAs and six DE mRNAs in MI and sham samples.

Enrichment analysis of differentially expressed mRNAs coexpressed by differentially expressed lncRNAs showed that the mRNAs were highly enriched in immune and inflammatory pathways, which was consistent with the pathogenesis of MI (Figure 3a and b). It is suggested that differentially expressed lncRNAs may interact with immune-related mRNAs and play an important role in MI.

To demonstrate which lncRNAs and mRNAs are involved in the immune pathway, the interactions among the lncRNAs and coexpressed mRNAs were first analyzed using Spearman correlation based on the RNA sequencing data. Annotation analysis of biological process pathways was conducted by differentially expressed lncRNAs expressed with differentially expressed mRNAs of MI compared with sham samples. According to the results, we found that a large number of the differentially coexpressed genes were primarily linked to the immune system process, inflammatory response and innate immune response (Figure 3c). Finally, 6 lncRNAs and 6 mRNAs of interest were selected to validate the reliability of the RNA-seq data (Figure 3d and e).

**Figure 4:**
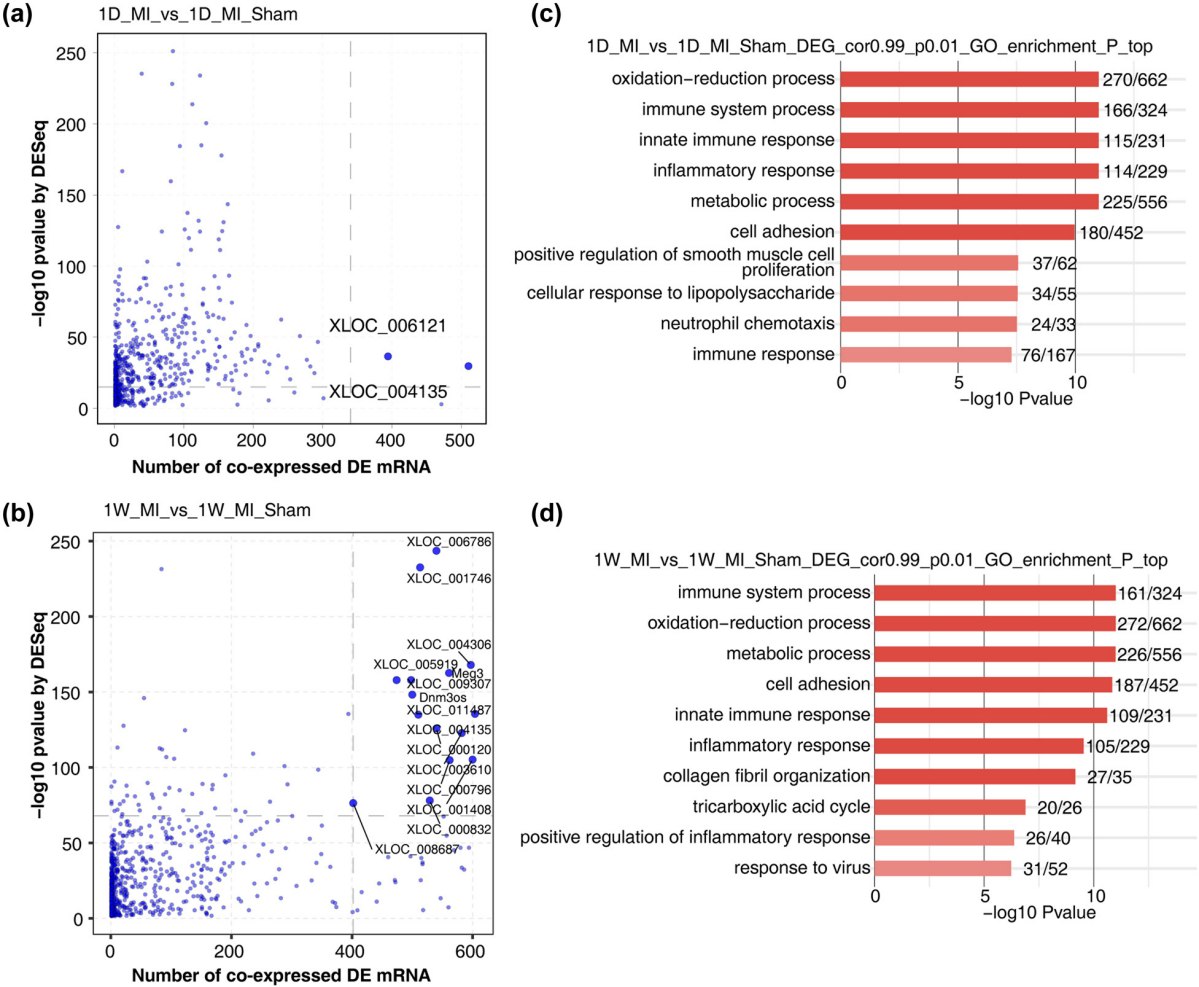
Coexpression network illustration between DE lncRNAs and DE mRNAs. (a and b) Scatter plot shows DE lncRNAs by MI compared with sham samples and the number of coexpressed DE mRNAs. Red points denote upregulated lncRNAs involved in coexpression pairs, and blue points denote downregulated lncRNAs. Cutoffs of *P*-value <0.01 and Pearson coefficient >0.99 were applied to identify the coexpression pairs; (c and d) top 10 most enriched GO biological process pathways by DE lncRNAs coexpressed with DE mRNAs of MI compared with Sham samples.

In addition, we used a scatter plot to show differentially expressed lncRNAs by MI compared with sham samples and the number of coexpressed differentially expressed mRNAs (Figure 4a and b). Then, the enriched GO biological process pathways were found to be mainly enriched in the immune and inflammatory pathways, as well as the very important oxidation‒reduction process in MI (Figure 4c and d).

### 3.5 Validation of the coexpression-related mRNA by qPCR

**Figure 5:**
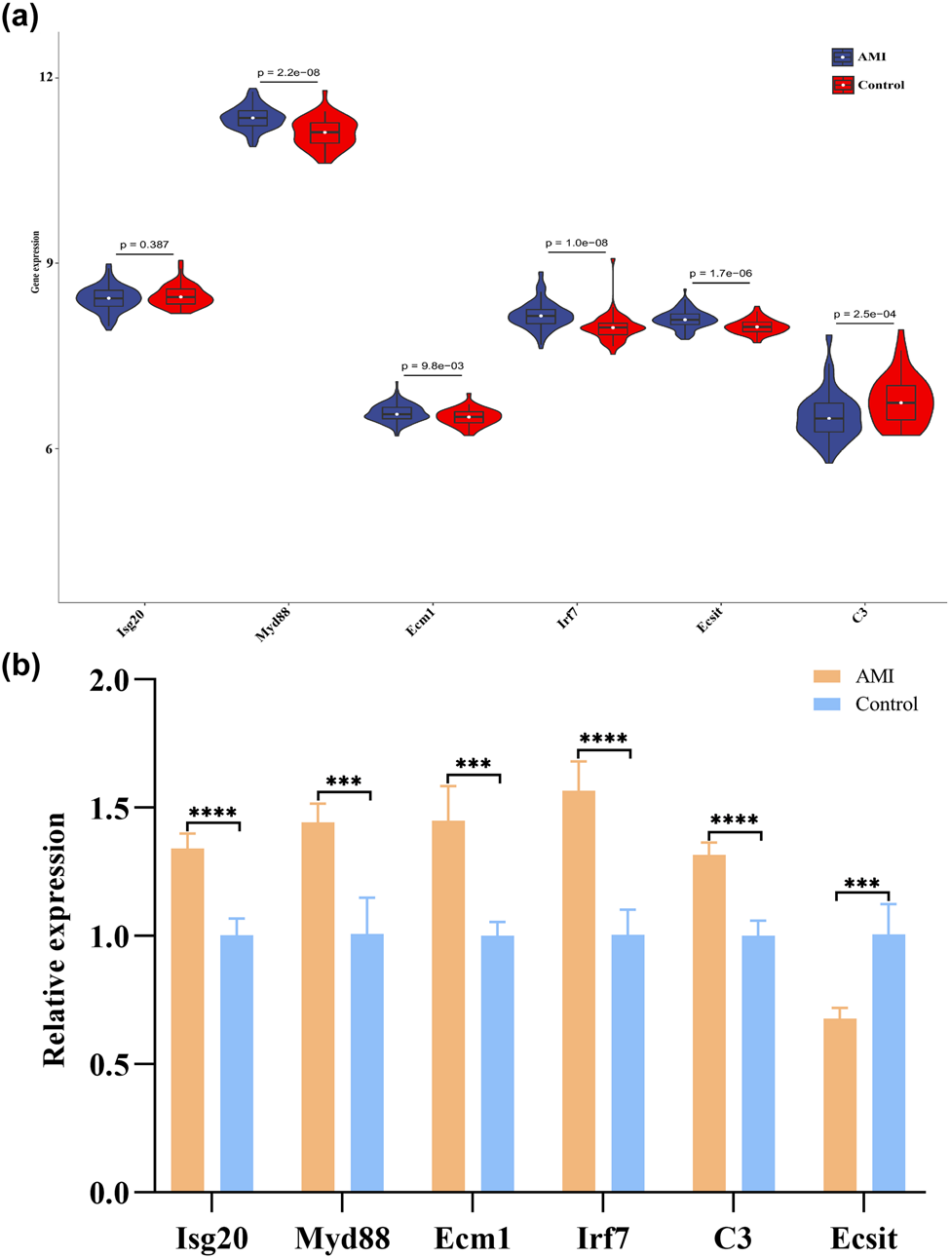
Expression of six highly coexpressed DE mRNAs was analysed. (a) The expression of the six coexpressed mRNAs in the GSE59867 dataset. (b) qPCR validation of the six coexpressed mRNAs between AMI and normal controls (^∗∗∗^ *P* < 0.001, ^∗∗∗∗^ *P* < 0.0001).

To validate the reliable expression changes of coexpression-related mRNAs in AMI, the GSE59867 dataset was used as an additional independent external validation set. Except for Isg20, the other five genes were differentially expressed in the two groups (Figure 5a), which also indicated that the coexpression-related mRNA may be consistent in mice and humans. Furthermore, blood samples from five normal and five AMI patients were collected, and the six highly coexpressed DEmRNAs in the coexpression network were further selected for qPCR validation. Compared with normal samples, the expression levels of Isg20, Myd88, Ecm1, Irf7, and C3 were increased, whereas those of Ecsit were decreased in AMI samples (Figure 5b). It was reported that Myd88, Ecm1, Irf7, Ecsit, and C3 were associated with MI, while Isg20, Irf7, and Ecsit were not directly related to MI but were related to inflammation. This suggests that differentially expressed lncRNAs may interact with immune-related mRNAs in MI.

### 3.6 WGCNA analysis of lncRNA‒mRNA coexpression modules

**Figure 6:**
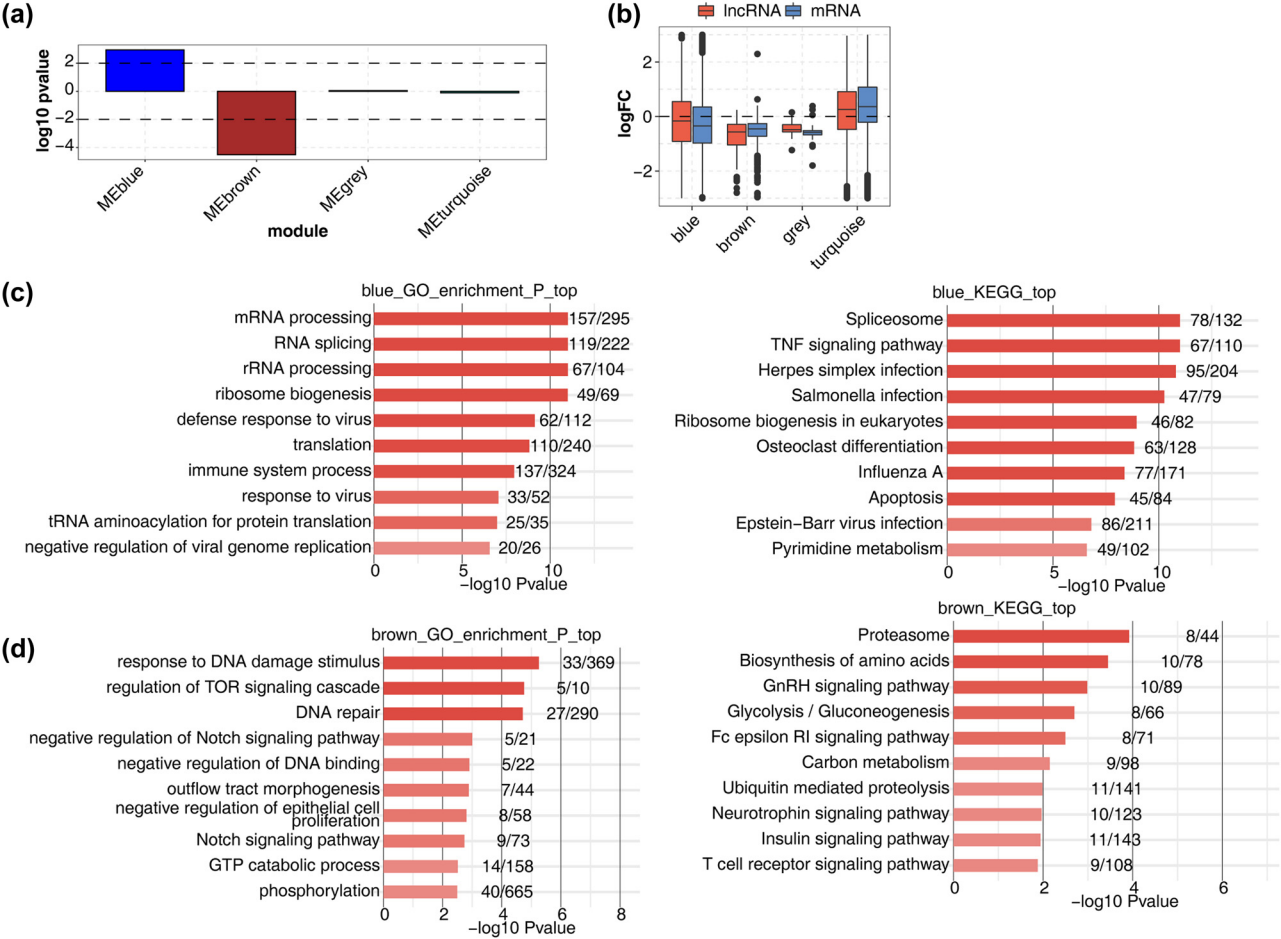
WGCNA of all expressed lncRNAs and mRNAs. (a) Signed association of module eigengenes with diagnosis of MI and Sham. Positive values indicate modules with increased expression in samples. Negative values indicate modules with decreased expression in samples. Dashed lines signify associated modules. (b) Boxplot showing expression fold change of mRNAs and lncRNAs from the four associated modules. The top 10 GO biological process and KEGG pathway enrichment of the modules MEblue (c) and MEbrown (d).

Through the gene expression module analysis of WGCNA, we found that two expression modules were highly associated with MI: MEblue and brown MEbrown (Figure 6a and b). Through GO biological process and KEGG pathway analysis, it was found that the functional pathway analysis of the MEblue module included immune system process, tumor necrosis factor signal pathway, and apoptosis (Figure 6c), while the functional pathway analysis of the MEbrown module included the T-cell receptor signal pathway (Figure 6d). The pathways involved in the two modules are also closely related to immunity.

### 3.7 *Cis*-acting analysis of lncRNA regulation

**Figure 7:**
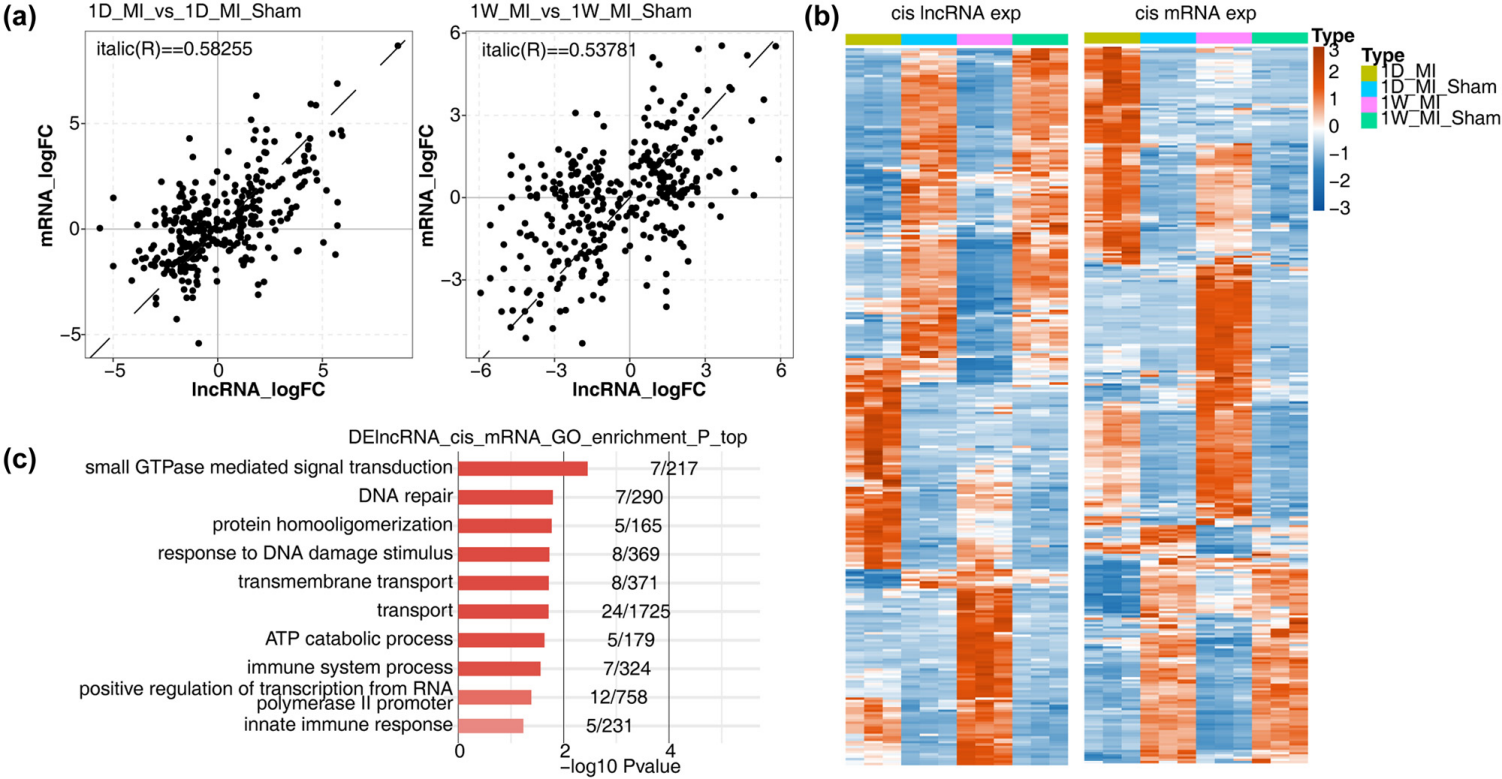
*Cis*-regulatory genes of DE lncRNAs. (a) Scatter plot shows log2 FC of DE lncRNAs by MI compared with Sham samples and its *cis*-regulatory genes. (b) Heatmap shows expression pattern of DE lncRNAs and its *cis*-regulatory genes. (c) Top 10 most enriched GO biological process pathways of *cis*-regulatory genes.

We analyzed the differentially expressed lncRNAs that may target the regulated differentially expressed mRNAs in MI. The results showed that *cis*-acting differentially expressed lncRNAs and mRNAs mainly showed a positive phase regulation relationship, which was visualized with a heatmap (Figure 7a and b). These differentially expressed mRNA-enriched functional pathways included immune system processes, innate immune responses, and the VEGF signaling pathway (Figure 7c).

## 4 Discussion

In the present study, we found the potential inflammatory role of lncRNAs in MI, which may help to further elucidate the mechanism of MI and provide potential targets for the diagnosis and treatment of acute MI. MI is a complex degenerative cardiovascular disease of concern due to its high morbidity and mortality [27]. In this study, the C57BL/6J mouse strain, one of the most widely used models for cardiovascular disease research, was selected as the MI animal model for RNA-seq analysis. First, lncRNA and mRNA expression profiles were determined using the hearts of 1-day-old and 1-week-old C57BL/6J mice. Then, many differentially expressed lncRNAs and mRNAs in MI were analyzed by RNA-seq. Cells and biological pathways in MI were then determined by GO, KEGG, and Pathnet analyses. Furthermore, coexpression networks revealed interactions between lncRNAs and mRNAs, and ceRNA networks were employed to show interactions between lncRNAs and miRNAs. Finally, the possible *cis*-regulated targets of differentially expressed mRNAs by differentially expressed lncRNAs in MI were explored.

We found that at 1 day or 1 week after MI, there was significant differential expression of lncRNAs after MI, and lncRNA PCA clustering found that the lncRNA expression profile could clearly distinguish between MI and non-MI samples. These lncRNAs can be used as potential diagnostic markers of MI. In fact, lncRNAs can stably exist in plasma, and peripheral blood is easy to obtain and easy to detect, so lncRNAs in circulating blood may become biomarkers for the diagnosis of acute MI (AMI). Li et al. [28] continuously monitored patients with acute ST segment elevation MI (STEMI). The results showed that compared with healthy volunteers, the expression of plasma lncRNA LIPCAR in patients with STEMI increased significantly within 4 h after the onset of STEMI symptoms, peaked at 12 h, and gradually returned to the baseline level on the 7th day. In addition, lncRNA LIPCAR showed the best sensitivity and specificity in the diagnosis of STEMI in the ROC curve, and there was a positive correlation between LIPCAR and Gensini score (coronary artery disease score), indicating that the increased degree of lncRNA LIPCAR reflected the severity of coronary artery stenosis [13]. Although these studies provide many potential new markers of MI, the sensitivity and specificity of these lncRNAs cannot be compared with the clinical markers of myocardial necrosis, and the study of lncRNAs still needs to be further explored.

In recent years, an increasing number of studies have shown that lncRNAs are related to the occurrence and development of MI [29,30,31], but the underlying mechanisms still need to be further elucidated. As regulators, lncRNAs are closely related to cellular inflammation [32,33], excessive reactive oxygen species [34], and apoptosis [35]. In addition, a large number of studies have shown that the immune and inflammatory responses after MI have an important impact on the prognosis of AMI patients [36,37]. In the context of MI, our study found abnormal expression levels of some inflammation- and immune-related genes, which may be potential targets of lncRNAs.

However, it is still unclear which lncRNAs are closely related to the occurrence and development of MI. In addition, the functions of most lncRNAs remain unclear to date. Currently, we predict lncRNA function according to their closely related coding genes. Combining bioinformatics analysis with literature validation, we found that differentially expressed mRNAs coexpressed with differentially expressed lncRNAs were highly enriched in immune and inflammatory pathways, which was consistent with the pathogenesis of MI.

Some studies have suggested that lncRNAs participate in the occurrence and development of MI. High mobility group protein 1 (HMGB1) is a ubiquitous nuclear protein. High mobility group protein 1 is a ubiquitous rich nuclear protein, and the expression of HMGB1 is significantly increased in injured myocardium during ischemia‒reperfusion. HMGB1 can induce the release of the inflammatory cytokines interleukin-6 and tumor necrosis factor-α, resulting in a severe inflammatory response. Shi et al. have shown that lncRNA TUG1 can reduce the expression of inflammatory factors by inhibiting HMGB1, thus reducing the inflammatory response of MI [38]. lncRNA H19, located near the telomere region of the human H19 gene on chromosome 11, is one of the most well-known imprinted genes. Increased expression of H19 can reduce cardiomyocyte apoptosis and inflammation, thereby reducing the dead area of MI, improving cardiac function, and reducing cardiac fibrosis. Previous studies have shown that lysine-specific demethylase 3A (KDM3A) is involved in myocardial ischemia‒reperfusion injury after MI by regulating key signaling pathways such as inflammation, apoptosis, and oxidative stress, and subsequent studies further confirmed that H19 regulates the expression of KDM3A through competitive binding to miR-22-3p, thus improving myocardial injury induced by MI [17].

Although these lncRNAs have been reported to regulate MI development, the roles of other lncRNAs remain to be further explored. We performed GO and KEGG pathway analyses, enriched the functions of differentially expressed lncRNAs and mRNAs by WGCNA, and identified important pathways involved in the occurrence and development of MI. The results revealed that immune and inflammatory pathways play an important role in the regulation of mRNAs by lncRNAs in MI mice. In fact, previous studies have shown that immune and inflammatory pathways are the canonical pathways in the occurrence and development of MI. This demonstrates that WGCNA is a significant pathway to find the mRNA pathway regulated by lncRNA.

Regarding the regulatory function of lncRNAs in immunity and inflammation, we constructed the lncRNA‒mRNA coexpression network and found that six differentially expressed lncRNAs (Gm26809, XLOC_008195, XLOC_000947, Gm12840, C130080G10Rik, XLOC_001747) and six differentially expressed mRNAs (Isg20, Myd88, Ecm1, Irf7, Ecsit, C3) are worthy of attention. Among them, Myd88 [39], Ecm1 [40], and C3 [41] were all reported to be related to MI, which verified previous studies: Isg20 [42], Irf7 [43], and Ecsit [44] were not reported to be directly related to MI but were closely related to immunity or inflammation. The role of these related differences between lncRNAs and mRNAs in MI needs to be further verified by further experiments.

Compared with protein-coding genes, most lncRNAs are less expressed and generally exhibit developmental stage- and tissue-specific expression [45,46,47]. Because of the high specificity and large number of lncRNAs expressed, they may be an important but untapped layer of regulatory information that determines the fate of cells during development [46,48]. The nonrandom location of lncRNAs implies a connection between lncRNA function and the genomic neighborhood [4,49,50]. However, whether lncRNA expression is associated with adjacent or distant protein-coding genes and whether lncRNAs preferentially regulate adjacent genes in *cis* have been controversial [51]. Several investigators now classify lncRNAs according to their genomic location relative to protein-coding sites and find that different lncRNAs have significant effects on the *cis* regulation of nearby transcription [49]. Therefore, in this study, through the enrichment analysis of *cis*-acting mRNAs of differential lncRNAs, the main pathways of enrichment included immune system process, innate immune response, VEGF, and other signal pathways.

Numerous studies have shown that innate immunity is activated after MI. Innate immunity both exacerbates ischemic injury and hinders remodeling after MI. In addition, activation of innate immune pathways in cardiomyocytes produces cytoprotective effects through mitochondrial stabilization, whereas activation that is more prolonged or of greater magnitude and involves immune cells results in more robust inflammatory responses and leukocyte recruitment, which aggravate myocardial injury [52]. However, innate immune activation contributes to myocardial healing and plays a crucial role in stable scar formation and prevention of intraventricular thrombosis after AMI.

Our study also has some limitations. On the one hand, the number of cases in the GSE114695 dataset was relatively small, and the samples were from mice, which may have some impact on the results of clinical MI. On the other hand, the study is descriptive, and further molecular experiments are needed to validate the data. We will further improve and supplement this work in future research.

## 5 Conclusion

In conclusion, we searched for lncRNA‒mRNA expression profiles in MI by RNA-seq and used bioinformatics analysis to analyze the underlying regulatory mechanisms. In this study, we aimed to reveal the potential inflammatory role of lncRNAs in MI, which may help to further elucidate the mechanism of MI and provide potential targets for the diagnosis and treatment of acute MI. However, our study also suffers from some limitations, such as the lack of experimental validation of the functions of these differentially expressed lncRNAs. More studies are needed to explore the role of these differentially expressed lncRNAs in MI.
